# Social Representations and Experiences of Sexual Transactions Among Swiss Youth

**DOI:** 10.1007/s10508-024-02814-8

**Published:** 2024-02-27

**Authors:** Annamaria Colombo, Myrian Carbajal, Riccardo Milani

**Affiliations:** School of Social Work, HES-SO, University of Applied Sciences and Arts Western Switzerland, Fribourg, 1700 Switzerland

**Keywords:** Transactional sex, Sexual transactions, Social representations, Youth, Heteronormativity, Switzerland

## Abstract

This article relies on quantitative data collected in Switzerland as part of a research study on sexual transactions among youth. Building on an analytical framework that defines sexual transactions in terms of negotiated exchanges rooted in social representations, we explored how they were perceived by the Swiss young people included in our sample at a cognitive, ethical, and political level. We found that research participants who reported having experienced sexual transactions viewed them much more positively than those who reported never having engaged in such exchanges. While this was especially true among young women, we also found that the tendency of respondents to perceive sexual transactions negatively increased with age. When analyzed in light of the qualitative results of our study, these quantitative findings suggest that negative representations of sexual transactions are less likely to be based on lived experience than on an ideal-type of sexual behavior. In other words, our research highlights how young people interpret sexuality according to norms developed within a heteronormative matrix.

## Introduction

This article relies on quantitative data collected as part of a research study titled “Sex, Relationships… and You? Sexuality and Sexual Transactions Involving Youth in Switzerland.” Conducted in three language regions between 2015 and 2017 (Colombo et al., 2017a), the study focused on sexual transactions, that is to say sexual experiences involving the exchange of money, material goods, and/or symbolic resources. A three-stage methodology consisting of an online survey, qualitative interviews with youth, and focus groups with professionals was designed to capture the views of young people aged 14–25, as well as those of experts and stakeholders in the fields of sexuality studies and sexual health. Youth experiences, as documented through the qualitative interviews, have been discussed in Colombo et al. (2017b, 2022), as well as in Colombo and Carbajal (2019). The perspectives of professionals on youth representations, practices, and needs have been analyzed in Carbajal and Colombo (2021, 2023). For its part, this article focuses on the results of the online survey.

Given the extent to which the literature currently focuses on risks associated with juvenile sexuality, the opening section argues for considering sexual transactions in terms of how young people represent and assign meaning to such practices. The second section outlines the study’s theoretical framework, whereas the third describes the methods we used to collect our quantitative data. Based on the findings presented in the fourth section, the fifth and final section discusses the results of our quantitative analysis in light of the qualitative data gathered through interviews with young people.

### Literature Review

Scholars writing in English have made extensive use of the terms “transactional sex” and “sexual transactions” to describe sexual relations in return for payment, especially cases involving adults in the Global South (see, for example, Bui et al., 2014; Makhakhe et al., 2017; Megaputri, 2020). By contrast, the French-language literature on sexuality rarely mentions *transactions sexuelles* outside of references to the work of Tabet (2004) and her concept of sexual-economic exchange, which is understood in terms of a continuum of market and non-market transactions (Broqua & Deschamps, 2014; Crevoisier & Donzallaz, 2022). The term prostitution is still widely used in the French-language literature to describe sex work (e.g., Lavaud-Legendre, 2022b).

In both languages, the concepts of transactional sex or sexual transactions are less likely to be applied in studies focused on youth, despite extensive international research published since the 1980s on children and young people who have sexual relations in return for payment, including numerous recent literature reviews (for example, Benavente et al., 2022). Since the 1990s, scholars have preferred to use the term “victim” (of sexual exploitation, child trafficking, etc.) when discussing juvenile sex work. Accordingly, youth tend to only be described as engaging in sexual transactions when they obtain material rewards (Averdijk et al., 2019; Fredlund et al., 2013; Homma et al., 2012; Lavoie et al., 2010; Leclerc-Madlala, 2003; van de Walle et al., 2012). Nevertheless, some of the researchers in question (Fredlund et al., 2013; Lavoie et al., 2010; Leclerc-Madlala, 2003) have highlighted the fact that many of the young people involved rely on sexual transactions for access to consumer goods, especially luxury goods, rather than for survival. Typified by an epidemiological approach, these studies aim to highlight factors that may lead youth to become involved in such exchanges, including a history of abuse (sexual, mental, or physical), a confluence of stressful events, mental health problems, a strained family environment, a problematic parent–child relationship, or a lack of social support (Fredlund et al., 2013; Lavoie et al., 2010; Svensson et al., 2013). They also stress associated psychological consequences—including shame, a fear of being discovered by family members, and the emotional impacts of sexual coercion (van der Walle et al., 2012)—as well as the risks of contracting sexually transmitted infections (Homma et al., 2012).

In Switzerland, several national surveys have addressed youth sexual behavior, including the Swiss Multicentre Adolescent Survey on Health, which focused on the lifestyles of individuals between the ages of 16 and 20 (SMASH; Narring et al., 2002); the Health Behavior in School-aged Children survey (HBSC; Delgrande Jordan et al., 2019); the Sexual Health and Behavior of Young People in Switzerland surveys (Barrense-Dias et al., 2018; Bodmer, 2009); and the Optimus study on the sexual victimization of children and adolescents in Switzerland (Averdijk et al., 2012). While providing data on the physical, mental, and sexual health of adolescents, including the impact of sexual violence, such research has generally overlooked the issue of sexual transactions. One notable exception is the study by Debons et al. (2019), which focuses on the LGBTIQ+ community. According to Debons et al., 13.1% of young people aged 24–26 who defined themselves as non-heterosexual reported having received financial, material, and/or symbolic rewards in exchange for sexual intercourse at least once in their lifetime (compared to 2.4% of heterosexuals). The same study found that the likelihood of having engaged in sexual transactions was higher among men, as well as among individuals experiencing homelessness or increased economic and social insecurity.

At the international level, the literature on young people’s sexual behavior is dominated by epidemiological approaches to identifying factors associated with “risky” sexual behavior (early sexuality, a high number of partners, unprotected sex, coercive relationships, etc.). For example, various researchers have established a link with the consumption of alcohol or psychoactive substances, abuse, and gang affiliation. This literature also emphasizes how different forms of risky behavior are mutually reinforcing, especially when peer pressure comes into play (Boislard et al., 2009; Ha et al., 2016; Lemelin et al., 2014; Ritchwood et al., 2015). And yet, other scholars have found that the consumption of alcohol or other substances can alter disinhibition and facilitate stress management, thereby improving sexual performance (Lemelin et al., 2014; O’Sullivan & Thompson, 2013; Young et al., 2007). A much smaller group of studies have looked at the meanings young people assign to risky sexual behavior, its importance as a form of rebellion against adult authority (Zimmermann et al., 2017), or as a sign of the need for targeted professional support (Svensson et al., 2013).

In short, most of the literature on youth sexual behavior assimilates sexual transactions to risky sex and therefore treats them as inherently dangerous. This reflects a paradigm shift in the study of juvenile sexuality that has been underway since the 1990s, with scholars becoming less likely to portray young people involved in (economic-)sexual transactions as “deviants” and more likely to consider them “young people at risk.” Taking an evidence-based and epidemiological approach, most of the relevant literature focuses on risk factors associated with sexual transactions along with the development of interventions (especially medical and psychological ones) capable of preventing such exchanges. Meanwhile, very few researchers have explored the understandings, judgments, and positioning of young people themselves in relation to their experience of such social practices, a situation that leaves little room for seeing sexual transactions as anything other than a danger that youth must be protected from, lest they become victims.

In addition to obscuring young people’s agency, this dominant view of sexual transactions also poses concrete problems for professionals who work with youth. Indeed, the results of our online survey indicate that the same outlook prevails in the fields of social work and health care (Carbajal & Colombo, 2021). As a result, professionals often find themselves at a loss when confronted with young people who invoke consent, individual freedom, and ownership of their own bodies to justify their involvement in sexual transactions (Lavaud-Legendre, 2022b).

Our review of the literature also found that most existing studies take an overly monolithic view of sexual transactions. For instance, they tend to only consider cases that involve both exchanges of money and penetrative sex. Meanwhile, little research has been conducted on norms and representations that influence the decision whether to engage in such exchanges, especially the predominance of heterosexist and cissexist representations of sexuality.

This article aims to help fill these gaps by exploring social representations of sexual transactions, while considering a range of sexual practices (i.e., not only penetrative sex) and types of exchange (i.e., not only economic or monetary transactions) involved. In analyzing these representations from the perspective of young people in Switzerland, we avoid making assumptions regarding the nature of sexual transactions (risky, gratifying, etc.). Moreover, we seek to understand how social representations of sexual transactions differ according to a young person’s age, gender identity, and emotional and sexual orientation—as well as to understand the relationship between such representations and experiences of sexual transactions.

### Theoretical Framework

Our study was not designed to assess the prevalence of sexual transactions among Swiss youth. Instead, we set out to better understand the meanings that young people give to these exchanges. And rather than imposing a normative definition of what constitutes a sexual transaction, we took a comprehensive approach that emphasizes the subjective logic and social norms surrounding how young people experience them. With these goals in mind, we adopted a theoretical framework that draws on the sociology of representations, gender and feminist studies, the negotiated sexuality approach, and the sociology of social transactions (hence our preference for “sexual transactions” over “transactional sex”). Insofar as we focus on young people’s social representations of sexual transactions, the framework is rooted in the sociology of representations and especially Jodelet’s (1989) work on how youth collectively name and define different aspects of everyday reality through processes of interpretation, self-regulation, and positioning. We operationalize the notion of social representation according to the analytical framework proposed by Parazelli et al. (2013), which is based on the work of Karsz (2004). In other words, we interpret how individuals behave and the purpose of their behavior in terms of the normative principles they use to explain specific situations. Such an approach makes it possible to apply the concept of social representation at a cognitive, ethical, and political level (see the next section for a detailed description of these three levels).

However, it is important to remember that heterosexist and cissexist representations of sexuality still predominate in the Swiss context. In the fields of gender and feminist studies, heterosexism is defined as a system of social representations that portray heterosexuality as more legitimate than other sexual orientations (Chauvin & Lerch, 2013; Dayer, 2014). Based on the idea that there are only two biological sexes that give rise to two profoundly different genders, heterosexist ideology holds that the male gender is superior to the female gender and that any sentimental and/or sexual relationship must involve two people of different genders (Dayer, 2014). As for cissexist ideology, it is rooted in a system of social representations based on the belief that cisgender identity is more legitimate than other gender identities (Chauvin & Lerch, 2013; Dayer, 2014). These two systems of normative representations serve the dual role of regulating and producing subjects (Butler, 1997). In other words, the representations in question create expectations of social behavior—including young people’s sexual behavior. However, we agree with Butler (1997) when she argues that agency allows people to adopt a range of attitudes, from normative reproduction to more subversive beliefs.

Our approach to sexuality is aligned with that of Gagnon and Simon (1973), who saw it as deeply rooted in social interactions. Accordingly, we understand sexual transactions in terms of “negotiated sexualities” (Broqua & Deschamps, 2014; Combessie & Mayer, 2013) and insights from the sociology of social transaction (Rémy et al., 1978; Schurmans, 2013). Drawing on the idea of a continuum of market and non-market transactions (Tabet, 2004; Zelizer, 1989), the concept of sexual transactions proposed by Broqua and Deschamps (2014) allows for the consideration of various types of exchange (financial, emotional, or simple recognition) and manifestations of sexuality (physical relationships as well as associated issues of seduction, [self-]regulation, and [self-]control). We therefore see sexual transactions as dynamic processes involving different ways that individuals “mutually adjust” their behavior in the context of social interaction (Schurmans, 2013, p. 88).

Within this theoretical framework, the article analyzes the dynamics involved in how youth negotiate sexual transactions, with an emphasis on the meanings young people ascribe to such exchanges. And while recognizing that sexual transactions can involve risk, power imbalances, and even domination, we believe it is important not to overlook other meanings and types of relationships that may be associated with such exchanges.

## Method

### Participants

In 2015, the HES-SO School of Social Work Fribourg surveyed a non-representative sample of Swiss youth aged 14 to 25 on sexuality, sexual experiences, and sexual transactions (Colombo et al., 2021). The minimum age was set based on the inclusion of sensitive questions related to intimacy and sexuality. Indeed, previous research had found that young people start to have a sufficient grasp of such topics at the age of 14 (Lynch et al., 2019; Sanci et al., 2005).

The research team designed and tested the questionnaire with input from field experts and young people. This ensured that the survey’s language closely matched that commonly used by youth. With a view to raising awareness, informing participants, and increasing participation, the team launched an extensive French-, German-, and Italian-language communication campaign using both traditional and digital media. Researchers developed dedicated websites, distributed posters and flyers, disseminated project-related information through their personal and professional networks (targeting organizations and agencies active in the fields of social work, community health, sexual health, sex work, juvenile justice, and youth recreation), and contributed content to local newspapers and radio stations. The final sample was based on a set of 7,657 completed questionnaires (out of a total of 8,624). Some 967 submissions were rejected because they were submitted from non-Swiss IP addresses or because the answers were incomplete, contradictory, non-serious, or otherwise unusable. Despite the significant level of participation, only about half of respondents answered the questions on sexual experiences and an even smaller proportion provided insights on sexual transactions. Given how response rates varied by question, the analytical sample retained for this article consists of 3,724 individuals, whose age and gender profile differs from that of the larger study sample. In particular, the analytical sample is more balanced in terms of gender (the proportion of young men went from 58 to 51%) and includes a lower proportion of late adolescents (31% compared to 39%). These differences suggest that men over the age of 18 often preferred not to answer the questions on sexual experiences, sexual transactions, and social representations of sexuality.

### Measures

#### Social Representations of Sexual Transactions

In the context of this article, social representations of sexual transactions constitute dependent variables. We operationalize the underlying concept based on the analytical framework proposed by Parazelli et al. (2013). As noted in the previous section, it covers three levels of normative criteria:

- Understandings (cognitive level): How do youth understand sexual transactions?
- Judgments (ethical level): What do youth consider acceptable or unacceptable in terms of sexual transactions?
- Positioning (political level): How do young people position themselves vis-à-vis sexual transactions?

#### Understandings of Sexual Transactions

The first set of dependent variables (cognitive level) relate to young peoples’ understandings of sexual transactions. The survey prompted respondents to assess the connection between such exchanges and various social contexts or characteristics that the media and public discourse often associate with youth socialization and sexuality: (1) nightlife and parties; (2) school; (3) Internet and smartphones (including social networks like Snapchat, Facebook, WhatsApp, and Instagram); (4) homosexuality; (5) sexy clothing; (6) drugs and alcohol; and (7) sex work. The selection and wording of these items was thoroughly discussed with the field experts and young people who assisted with designing the questionnaire. The proposed associations intentionally incorporated stereotypical (and sometimes contested or even stigmatized) representations of youth and their behavior that frequently appear in the media and popular culture. This approach was intended to provide research participants with the opportunity to express their agreement or disagreement with such associations. For example, the terminology used to describe sex work was aligned with youth vernaculars prevalent in the different linguistic regions of Switzerland: *prostitution* in French, *Prostitution* in German, and *Prostituzione* in Italian. To reiterate, these word choices were intended to closely mirror how young people speak and do not reflect the authors’ theoretical orientations.

Research participants were asked to rate each item’s relevance to sexual transactions on a scale from 1 (“not at all relevant”) to 3 (“very relevant”). Table 1 summarizes the results for all seven items. Later in the article, we analyze the correlation between experiences of sexual transactions and the three most frequently associated contexts or characteristics—i.e., sex work, Internet and smartphones, and drugs and alcohol.

**Table 1:** Understandings of sexual transactions

Social contexts or characteristics	Obs	Mean	SD	Min	Max
Sex work	4,387	2.468	0.747	1	3
Internet and smartphones	4,386	2.380	0.704	1	3
Drugs and alcohol	4,386	2.356	0.721	1	3
Nightlife and parties	4,388	2.165	0.730	1	3
Sexy clothing	4,386	2.029	0.762	1	3
School	4,386	1.645	0.700	1	3
Homosexuality	4,386	1.381	0.617	1	3

Source: Based on Colombo et al. (2021)

#### Judgments in Relation to Sexual Transactions

The second set of dependent variables (ethical level) relate to judgment. Accordingly, the survey asked respondents for their reactions to a series of prescriptive propositions. In other words, we asked research participants whether they agreed or disagreed with a series of statements on sexual transactions:

1. There’s nothing wrong with offering something in exchange for sex.
2. There’s nothing wrong with accepting something in exchange for sex.
3. It’s better to offer or accept a gift than money.
4. It’s less of a problem if the man offers something in exchange for sex.
5. It’s less of a problem if the woman accepts something in exchange for sex.

The statements were worded in such a way as to prompt young people to address the binary conception of gender promoted by the prevailing heterosexist and cissexist ideologies reflected in social representations.

Respondents could either agree or disagree with each statement. Based on the responses obtained and the correlations between the different prescriptive propositions, we generated three variables for analysis. First, we aggregated the first two statements (*r* = 0.65, *p* < 0.001) to create a single dichotomous variable in cases where both received an affirmative answer. Second, we did the same with the last two statements (*r *= 0.47; *p* < 0.001). Finally, we decided to analyze responses to the third statement (“It’s better to offer or accept a gift than money”) separately. Table 2 summarizes the results for each individual item and the three retained variables.

**Table 2:** Judgments of sexual transactions

Ethical assessments	Obs	%
Nothing wrong with offering something in exchange for sex	4,980	16.4
Nothing wrong with accepting something in exchange for sex	4,979	15.2
Better to offer or accept a gift than money	4,976	38.6
Less of a problem if the man offers something	4,976	17.6
Less of a problem if the woman accepts something	4,974	13.0
Appropriate for men or women to offer/accept	4,981	11.2
Better to offer/accept a gift than money	4,976	38.6
Less of a problem for the man to offer/the woman to accept	4,975	8.3

Source: Based on Colombo et al. (2021)

#### Positioning vis-à-vis Sexual Transactions

The third and final set of dependent variables (political level) relate to how respondents positioned themselves when asked to judge sexual transactions in terms of normality, goodness, dominance, justice, pleasure, social acceptance, etc. In each case, research participants used a slider to indicate more or less positive attitudes according to a differential semantic scale. We retained four variables for analysis, including the three pairs with the lowest mean: (1) humiliating-respectful, (2) bad-good, and (3) abnormal-normal. We also created a composite indicator (negative–positive) to aggregate the scores for all pairs through principal component analysis. Table 3 summarizes the results for the various pairs as well as the composite indicator.

**Table 3:** Positioning vis-à-vis sexual transactions

Semantic scales	Obs	Mean	SD	Min	Max
Humiliating-respectful	4,146	18.65	24.36	0	100
Bad-good	4,356	19.10	25.85	0	100
Abnormal-normal	4,514	19.22	27.59	0	100
Risky-safe	4,126	20.28	23.96	0	100
Weak-strong	3,753	22.64	29.30	0	100
Unpleasant-pleasant	3,874	26.86	29.90	0	100
Violent-tender	3,504	27.36	25.99	0	100
Selfish-generous	3,579	27.53	29.64	0	100
Unfair-fair	3,610	29.25	30.67	0	100
Uncool-cool	3,366	34.66	33.58	0	100
Submissive-empowering	4,064	36.82	34.91	0	100
Passive-active	3,087	37.13	34.73	0	100
Forbidden-allowed	3,917	43.38	36.19	0	100
Negative–positive*	1,716	24.87	25.13	0	100

*N is lower because the “negative–positive” index is a composite measure that does not account for missing values in the original variables. This is why we also chose to analyze three of the original differential scales. Source: Based on Colombo et al. (2021)

#### Experiences of Sexual Transactions

“Sexual transactions” refers to sexual experiences involving the exchange of money, material goods, or symbolic resources. Whether a respondent had experienced sexual transactions constitutes one of the study’s independent variables. However, to avoid imposing an a priori definition and in recognition of the concept’s multidimensional nature, the study questionnaire referred to “sexual experience(s) in exchange for something.” In this way, respondents were free to associate sexual transactions with the receipt of gifts (a telephone, cigarettes, clothes, drugs, money, etc.) or status (being accepted into a group, improving their reputation, etc.). In addition, this methodological choice allowed for consideration of a range of sexual practices, beyond just sexual intercourse. Accordingly, we established four categories of behaviors associated with sexual transactions: (1) kissing or petting, (2) showing private body parts (in person or by sending nude images), (3) oral sex, and (4) sexual intercourse.

#### Demographics

The data gathered as part of our research study allow for exploring the relationship between experiences and social representations of sexual transactions in terms of various personal characteristics. For this article, we have retained three variables.

First, we divided the sample into men (0) and women (1), based on self-reported gender. Because the question on gender identity used in the 2015 survey only offered these options, other gender identities could not be reliably determined. While we acknowledge the limitations of this approach and the ongoing methodological debate on how best to collect data on gender identity (see, for example, Lindqvist et al., 2021), issues with the original question have little impact on the potential for quantitative analysis. Especially in smaller samples, individuals not identifying strictly as man or woman typically represent a small percentage of respondents (often below 5%) and are usually excluded from quantitative analyses (Medeiros et al., 2020).

Second, we divided the sample into three age groups based on the different stages of adolescence: early adolescents (ages 14–16), mid-adolescents (ages 17–18), and late adolescents (ages 19 +).

Finally, we accounted for respondents’ self-reported emotional and sexual orientations, identifying those who reported opposite-sex attraction as 1, those who reported same-sex attraction as 2, and those who reported being attracted to both sexes as 3. We excluded other emotional and sexual orientations due to a lack of observations. Table 4 summarizes the distribution of demographic characteristics and experiences with different categories of sexual transactions within the analytical sample.

**Table 4:** Categories of sexual transactions and personal characteristics (analytical sample)

Variable	Obs	%
Experience of sexual transactions	3,724	15.7
Kissing or petting	3,669	9.6
Showing private body parts	3,190	10.9
Oral sex	2,828	11.1
Sexual intercourse	2,741	10.3
Gender	4,387	
Man	2,247	51.2
Woman	2,140	48.8
Age group	4,387	
14–16	1,356	30.9
17–18	1,657	37.8
19 +	1,374	31.3
Emotional and sexual orientation	4,133	
Opposite-sex attraction	3.410	82.5
Same-sex attraction	227	5.5
Attraction to both sexes	496	12

Source: Based on Colombo et al. (2021)

### Statistical Analysis

Given the focus on the cognitive, ethical, and political dimensions of social representations, our empirical strategy relied on three distinct econometric specifications to estimate the correlation between respondent characteristics and the representations they embraced. This allowed us to assess the moderating effect of having experienced sexual transactions on the relationship between individual characteristics and social representations.

First of all, we applied a multivariate ordered probit regression model to the three discrete variables associated with the cognitive level (sex work, drugs and alcohol, and Internet and smartphones). This made it possible to jointly model different dependent variables, thereby accounting for the theoretical correlation between the residuals of different social constructs retrieved from the same survey population (*p* < 0.001).

Following a similar approach, we found that the three dichotomous variables associated with the ethical dimension (“Appropriate for men or women to offer/accept,” “Better to offer/accept a gift than money,” and “Less of a problem for the man to offer/the woman to accept”) are also positively correlated (*p* < 0.001). In this case, we opted for a multivariate probit regression with robust standard errors to achieve a more reliable parameters estimation.

With respect to the political dimension, we accounted for overdispersion in each of the three dependent variables (humiliating-respectful, bad-good, and abnormal-normal) by applying a negative binomial regression model to the relationship between positioning and experiences of sexual transactions. In the case of the composite indicator (negative–positive), we did so using linear regression.

For all nonlinear models (cognitive, ethical, and political levels), we measured interaction effects using second derivatives, as proposed by Mize (2019). We then replicated the analyses by substituting the “experience of sexual transactions” variable with variables corresponding to distinct sexual behaviors (sexual intercourse, oral sex, showing private body parts) to test for variations. These additional results are presented in Appendix.

## Results

The results of our analysis reveal that respondents who reported having experienced any category of sexual transactions (referred to below as respondents “with experience”) were much more likely to take a positive view of such exchanges than those without experience. However, the correlation appears to decrease with age. In any case, these findings were reflected in young peoples’ understandings (cognitive level), judgments (ethical level), and positioning (political level) in relation to sexual transactions.

### How Did Respondents Understand Sexual Transactions?

As shown in Table 1, research participants mainly associated sexual transactions with sex work, Internet and smartphones, and drugs and alcohol—as opposed to more everyday social contexts like school. And although the ordered probit regression analysis on these three dependent variables (Table 5) suggests that young people strongly associate sexual transactions only with sex work, the association between sex work and such exchanges was lower among respondents with experience. Nor did the latter group associate sexual transactions with drugs and alcohol or Internet and smartphones when considering all categories combined. However, responses regarding exchanges specifically involving sexual intercourse or showing private body parts suggest a positive correlation with Internet and smartphones (see Table 8). In addition, woman research participants were more likely to associate sexual transactions with all three social contexts. However, those with experience were less likely to associate them with sex work. Older respondents were more likely to view sexual transactions negatively and associate them with sex work, drugs and alcohol, and the Internet and smartphones. By contrast, these associations were less pronounced among younger respondents. With respect to emotional and sexual orientation, the results are less clear. Research participants attracted to both sexes associated sexual transactions with sex work and drugs and alcohol less often than those attracted to the opposite sex. However, those with experience were more likely to associate such exchanges with sex work. Generally speaking, the results are consistent even when the different categories of sexual transactions are considered (see Table 8 in Appendix).

**Table 5:** Correlations between experiences and understandings of sexual transactions

	Social contexts		
	Sex work OR (Rob. SE)	Drugs/alcohol OR (Rob. SE)	Internet/smartphones OR (Rob. SE)
Experience of sexual transactions	0.480*** (0.052)	0.894 (0.097)	1.207 (0.135)
Gender (woman = 1)	1.342*** (0.060)	1.286*** (0.053)	1.187*** (0.050)
Mid-adolescent	1.181** (0.066)	1.179** (0.061)	1.135* (0.059)
Late adolescent	1.236*** (0.070)	1.127* (0.060)	1.317*** (0.071)
Same-sex attraction	1.004 (0.102)	0.841 (0.078)	0.917 (0.090)
Attraction to both sexes	0.858* (0.062)	0.784*** (0.052)	0.980 (0.070)
Interactions (second derivatives)	**∆2**	**∆2**	**∆2**
Sexual transactions*woman	− 0.122**	− 0.054	− 0.051
Sexual transactions*mid-adolescent	0.121**	0.041	− 0.042
Sexual transactions*late adolescent	0.240***	0.085	− 0.046
Sexual transactions*same-sex	− 0.091	− 0.132	− 0.034
Sexual transactions*both sexes	0.138**	0.052	0.075
Observations	3710	3710	3710

Multivariate ordered probit regression with moderated effects. Odd ratios with robust standard errors in parentheses. 2Δ = second differences for interactions terms (first differences are omitted for brevity). The estimates for men, early adolescents, and opposite-sex sexual attraction are not shown in the regressions due to perfect collinearity. *** *p* < 0.001; ** *p* < 0.010; * *p* < 0.050

### How Did Respondents Judge Sexual Transactions?

Table 6 shows the results of multivariate probit regression on the three ethical assessments. The findings suggest that while most respondents considered sexual transactions problematic, those with experience did not share this view. Indeed, young people who have been involved in such exchanges appear more likely to see nothing wrong with offering or accepting something in exchange for sex. The same group tended to see gifts as a less problematic form of compensation than money, and sexual transactions as less problematic when men offer compensation and women accept. With respect to gender, differences in the judgments expressed by women and men also varied depending on experience. Compared to young men, young women without experience were more likely to judge sexual transactions negatively, whereas women with experience took a more positive view—especially in the case of exchanges involving gifts rather than money. In terms of age, mid- and late adolescents with experience judged sexual transactions less favorably than early adolescents. The latter group considered it inappropriate to either offer or accept something in exchange for sex. Judgments also varied according to respondents’ emotional and sexual orientations, with those attracted to both sexes seeing sexual transactions as appropriate regardless of who offers or accepts compensation. Finally, the findings show no statistical differences by category of sexual transaction (see Table 9 in Appendix).

**Table 6:** Correlations between experiences and judgments of sexual transactions

	Ethical assessments		
	Appropriate for men or women to offer or accept OR (Rob. SE)	Better to offer/accept a gift than money OR (Rob. SE)	Less of a problem for the man to offer/the woman to accept OR (Rob. SE)
Experience of sexual transactions	3.151*** (0.420)	1.844*** (0.226)	2.098*** (0.298)
Gender (woman = 1)	0647*** (0.048)	0.756*** (0.035)	0.768** (0.050)
Mid-adolescent	1.005 (0.206)	0.953 (0.055)	0.929 (0.083)
Late adolescent	0.995 (0.094)	0.750*** (0.045)	0.716** (0.072)
Same-sex attraction	0.966 (0.158)	0.758* (0.084)	1.013 (0.169)
Attraction to both sexes	1.433** (0.153)	0.973 (0.078)	1.034 (0.131)
Interactions (second derivatives)	**∆2**	**∆2**	**∆2**
Sexual transactions*woman	0.044	0.099*	0.020
Sexual transactions*mid-adolescent	− 0.141**	− 0.094	− 0.081*
Sexual transactions*late adolescent	− 0.184**	− 0.121*	− 0.071
Sexual transactions*same-sex	− 0.012	0.012	− 0.069
Sexual transactions*both sexes	0.105*	− 0.088	− 0.010
Observations	3710	3710	3710

Multivariate probit regression with moderated effects. Odd ratios with robust standard errors in parentheses. 2Δ = second differences for interactions terms (first differences are omitted for brevity). The estimates for men, early adolescents, and opposite-sex sexual attraction are not shown in the regressions due to perfect collinearity. *** *p* < 0.001; ** *p* < 0.010; * *p* < 0.050

### How Did Respondents Position Themselves vis-à-vis Sexual Transactions?

The results indicate that, compared to respondents without experience, those with experience rated sexual transactions much more positively on the semantic differentials (Table 7). Overall, they considered them examples of normal, good, and respectful behavior. In terms of gender, although young women with experience were more likely to give negative ratings than young men with experience, their assessments were more positive than those of men who had never engaged in sexual transactions. Furthermore, age correlated negatively with a tolerant attitude toward sexual transactions, with mid- and late adolescents with experience holding the most stringent positions. Accordingly, older respondents were most likely to perceive sexual transactions as abnormal, bad, and humiliating. These findings are consistent with the results regarding cognitive and ethical perceptions. Finally, compared to research participants attracted to the opposite sex, respondents attracted to both sexes were more likely to take positive positions vis-à-vis sexual transactions, seeing them as normal and good. In this case, experience appears to have had no impact on the ratings given on the semantic scales. Once again, the results do not vary by category of sexual transaction (see Table 10 in Appendix).

**Table 7:** Correlations between experiences of sexual transactions and positioning vis-à-vis such exchanges

	Semantic scales			
	Negative–positive Coeff (Rob. SE)	Abnormal-normal IRR (Rob. SE)	Bad–good IRR (Rob. SE)	Humiliating–respectful IRR (Rob. SE)
Sexual transactions	37.97*** (4.037)	2.811*** (0.243)	2.715*** (0.232)	2.445*** (0.227)
Gender (woman = 1)	− 10.34*** (1.104)	0.535*** (0.030)	0.578*** (0.029)	0.568*** (0.028)
Mid-adolescent	− 6.29*** (1.580)	0.740*** (0.051)	0.731*** (0.046)	0.816** (0.051)
Late adolescent	− 7.71*** (1.533)	0.692*** (0.048)	0.659*** (0.042)	0.704*** (0.044)
Same-sex attraction	0.240 (3.111)	1.191 (0.135)	1.249* (0.131)	1.019 (0.108)
Attraction to both sexes	2.290* (1.811)	1.242* (0.106)	1.305*** (0.098)	0.979 (0.081)
Interactions	Coeff	**∆2**	**∆2**	**∆2**
Sexual transactions*woman	− 0.179	1.006	− 1.873	− 1.144
Sexual transactions*mid-adolescent	− 10.76*	− 12.28**	− 13.06**	− 15.36***
Sexual transactions*late adolescent	− 20.68***	− 23.65	− 22.73***	− 18.99***
Sexual transactions*same-sex	7.930	1.730	− 1.445	8.642
Sexual transactions*both sexes	12.28*	7.270	7.811	7.275
Observations	1334	3536	3419	3306

Note: Linear regression with moderated effects for Model 1 (negative–positive). Multivariate negative binomial regression with moderated effects for Model 2 (abnormal–normal), 3 (bad–good), and 4 (humiliating–respectful). Regression coefficients with robust standard errors in parentheses for Model 1. Incidence rate ratios with robust standard errors in parentheses for Models. 2Δ = second differences for interactions terms in nonlinear models (first differences are omitted for brevity). The estimates for men, early adolescents, and opposite-sex sexual attraction are not shown in the regressions due to perfect collinearity. *** *p* < 0.001; ** *p* < 0.010; * *p* < 0.050

### Summary of Key Results

Four main findings emerge from our quantitative analysis of the study data.

First, we found a positive correlation between experience with sexual transactions and more favorable social representations of such exchanges, regardless of whether the assessment was made at a cognitive, ethical, or political level. For instance, respondents with experience refrained from associating sexual transactions with socially stigmatized behaviors (e.g., sex work). Furthermore, they judged such exchanges positively and took a favorable stance toward them.

Second, representations of sexual transactions differ according to a young person’s gender. Specifically, the understandings, judgments, and positioning of female responents tended to be more negative than those of their male counterparts. But compared to young women without experience, those with experience generally expressed more positive views. In a single instance—young women with experience who associated sexual transactions with sex work—assessments were more positive than young men with experience. Otherwise, female respondents strongly rejected this association and even those with experience viewed sexual transactions less favorably than their male counterparts with experience.

Third, youth tend to view sexual transactions more negatively as they get older. Among our research participants, this trend was most pronounced in the case of respondents with experience.

Finally, compared to their heterosexual and homosexual counterparts, bisexual youth tend to embrace more positive social representations of sexual transactions. However, the results of our analysis were somewhat mixed in this regard. For instance, although respondents with experience who reported being attracted to both sexes were more likely to associate sexual transactions with sex work, they considered such exchanges acceptable provided the interaction was mutual (and regardless of whether the person offering or accepting something in exchange for sex was man or woman).

## Discussion

Below, we discuss these quantitative findings in the context of the qualitative results that have come out of the larger study. The methods used to collect qualitative data provided our young research participants with opportunities to define themselves more freely and comprehensively, rather than in relation to predefined categories such as those based on gender identity or emotional and sexual orientation. We begin by summarizing key outcomes of the qualitative interview analysis, before using them to contextualize the results presented above. Finally, we discuss avenues for future research.

### Overview of Qualitative Results

The second stage of the study involved conducting semi-structured interviews with 37 young people between the ages of 14 and 25 who had experienced sexual transactions. Twenty-one of these interviewees were from French-speaking Switzerland, nine were from a German-speaking region, and seven from the Italian-speaking part of the country. Eighteen of them identified as woman, 17 as man, and two as androgynous or mixed gender. Additionally, 24 of them identified as heterosexual, 11 as homosexual, one as bisexual, and one as pansexual. Most were recruited through the online survey, which included an option for respondents interested in participating in an interview to provide their contact information without compromising the anonymity of their survey responses.

Interviewees reported having experience with a wide range of sexual transactions, in terms of both the practices involved (kissing, caressing, oral sex, penetrative intercourse) and the benefits exchanged. Examples of such financial, material and/or symbolic rewards include a drink, a place to stay, money, intimate digital content, or acceptance into a group. Only a minority of interviewees clearly stated that they engaged in sex work (Colombo et al., 2022). Indeed, most of the experiences they described were more complex and nuanced than a commercial transaction between a person offering sexual services and another providing remuneration at a pre-negotiated rate. Instead, negotiations tended to take place in the context of sexual socialization and often according to a logic of recognition (Colombo et al., 2017b) and/or indebtedness (Carbajal et al., 2019). A large majority of the experiences in question were positive for the young people involved, insofar as they contributed to the construction of an emerging adult identity. Accordingly, they were associated with processes of sexual socialization, individual exploration, peer recognition, self-affirmation, and self-esteem (Colombo & Carbajal, 2019; Colombo et al., 2017b). But while interviewees spoke in terms of positive experiences, they were well aware of negative social representations of sexual transactions rooted in the association with sex work. For this reason, most of the young people we interviewed chose to keep these experiences secret (Colombo et al., 2017b, 2022).

### Analysis

These qualitative results help explain why the results of the quantitative survey show that young people with experience embrace more favorable social representations of sexual transactions than those without experience. They also highlight the need to interpret the responses of research participants with and without experience differently. Specifically, young people with experience can draw on direct knowledge of sexual transactions, which often involve positive experiences as well as a broad range of behaviors and forms of exchange (i.e., not simply someone offering sexual services in exchange for money).

As for young people without experience, who made up the majority of our quantitative sample, their main points of reference are the prevailing association of sexual transactions with sex work and the negative image of the “whore.” But as the qualitative results show, such social representations only correspond to a small segment of the sexual transactions actually experienced by young people. Accordingly, the responses of research participants without experience were generally based on an ideal-type representing “bad sexuality,” as opposed to lived experience or social realities. And as Zelizer (1989) has shown, sexual transactions tend to be perceived as antithetical to “proper” sexuality because of their potential to disturb affections and corrupt relationships by mixing intimacy with economic calculation. Likewise, those respondents without experience identified this mode of “deviant” sexuality with sexual behaviors—and, by extension, forms of love and intimacy—likely to undermine a young person’s respectability. In other words, young people typically embrace negative social representations of sexual transactions as a means of demonstrating their ability to meet social expectations governing sexual behavior.

Of particular interest is how these results highlight young people’s normative reference points for sexuality. Indeed, despite an apparent liberation of sexual mores, studies show that, in contemporary Western society, sexuality remains governed by strict normative expectations (Bozon, 2012; Clair, 2012). And far from being “immoral,” most of our respondents referred to a strict sexual morality. At a time when these young people were building their adult identities, it was particularly important for them to show adults and peers that they had fully assimilated prevailing social norms of behavior, especially with respect to sexual matters.

In terms of sexual morality, the qualitative results underscore the importance of respectfulness, which is strongly associated with the notion of consent, as well as pleasure and affectivity (Colombo et al., 2022). And because they are not always consensual and are often seen as devoid of affectivity and pleasure, sexual transactions constitute a transgression of sexual morality due to the absence of respect. Our interviewees expressed particular concern that sexual behavior be respectful toward women.

Such concerns reflect the perceived need to regulate women’s sexuality within a heteronormative matrix, where heterosexuality is the norm and the sexual roles of men and women are understood as different, complementary, and asymmetrical (Butler, 2006 [1990]). This leads to not only the subordination of female sexuality to male sexuality, but also the marginalization of non-heterosexual sexualities. Whereas male sexuality is associated with virility, sexual desire, and physiological needs, woman sexuality is associated with relationality, affectivity, and conjugality (Bajos et al., 2008; Déroff, 2007). From such a perspective, sexual transactions represent the opposite of woman sexuality and serve as a foil for young women seeking respectability (Colombo et al., 2017b). As they experiment with sexuality and seduction, they need to avoid gaining a reputation as a “whore” or a “slut”—figures emblematic of the transgression of sexual norms. Several of our interviewees referred to the “whore stigma,” which Pheterson (1993) has shown to be a burden on all women, not just those involved in sex work. It primarily serves to discourage women from acting contrary to social expectations.

Our analysis therefore reveals the extent to which young people are aware of social expectations informed by sexual scripts. Building on the theoretical work of Gagnon and Simon (1973), Bozon and Giami (1999) have described such scripts as defining “what is possible in terms of our sexuality.” Specifically, our results show how heteronormative sexual scripts play a central role in shaping cultural and social understandings of sexuality, serving to underpin the association between sexual transactions and sex work, and leading young people who have not experienced such exchanges to overwhelmingly reject them. In other words, when understood as financial arrangements, sexual transactions stand in stark contrast to forms of sexuality associated with respect, pleasure, and emotion. As for the especially strong opposition among young women to sexual transactions associated with sex work, it can be understood as a form of self-protection. Compared to young men, young women are more exposed to negative judgments regarding sexual behavior and therefore feel a greater need to prove their respectability. This makes them more inclined to demonstrate adherence to the norms governing “proper” sexuality (Mercier, 2018).

But even if female sexuality is much more closely policed than male sexuality, our qualitative results show that young men who identify as homosexual sometimes face constraints similar to those encountered by young women (Colombo et al., 2022). According to our interviewees, gay men are occasionally called “whore” or “slut.” And once again, these labels serve to control an individual’s sexuality. As Clair (2012) has shown, men tend to associate homosexuality with the feminine gender, because of how it challenges heteronormative constructions of virility. This could explain why the data collected from questionnaire respondents who reported same-sex attraction align more closely with that collected from respondents who identified as heterosexual (especially young men) than with the responses of research participants who identified as bisexual (especially young women). However, such conclusions would need to be refined through further research, given that we collected only limited data on gender identity as well as on emotional and sexual orientation.

Finally, our results show that an attachment to “proper” sexuality and a desire to meet the associated social expectations increases with age. As the literature suggests, adolescents tend to defy social rules as a means of breaking with the parental and social order, and building their own adult identity (Félonneau & Lannegrand-Willems, 2005). In this respect, young people expressing approval of behaviors that adults find troubling is far from unexpected and can be understood as part of the pathway from childhood to adulthood (Coslin, 2002). However, as young people mature and enter late adolescence, they become progressively more conformist, taking greater care to follow social and legal norms (Galland, 2003; Kohlberg, 1969). It is therefore not surprising that, compared to older respondents, younger research participants (ages 14–16) were somewhat more tolerant (or at least less vehement in their rejection) of sexual transactions—exchanges that, as we have seen, most respondents associated with the transgression of social norms.

### Avenues for Further Research

We believe that a better understanding of the norms that govern youth sexuality would make it possible to better support them through their sexual experiences and help them avoid associated risks. Our analysis has shown how gender, age, and (to a lesser extent) emotional and sexual orientation play an important role in young people’s relationship to sexuality. However, the underlying research study has not generated sufficient data to explore issues facing young people who do not identify as either man or woman. Other studies have found that these populations can be subject to discrimination, especially in matters of sexuality. Likewise, our analysis has explored emotional and sexual orientation in a relatively limited and binary way (opposite-sex attraction, same-sex attraction, attraction to both sexes). These dimensions of youth sexuality merit further investigation, especially with a view to understanding gender as well as emotional and sexual orientation in non-binary terms.

Other avenues for further research include how educational and career pathways correlate with normative representations of sexuality. Unfortunately, the relevant data collected in the context of our study are insufficiently differentiated and do not allow for a detailed analysis. Furthermore, our focus on Switzerland—a geographically small country with few large urban centers—has prevented us from clearly establishing correlations between socialization in an urban or rural context and representations of sexuality. Studies conducted in other national contexts may be able to shed some light on this subject. Finally, both the quantitative and qualitative results of our study highlight the importance for young people of having a trusted adult with whom they can talk about sexuality (Colombo et al., 2017a, 2021). Researchers should seek to clarify this role as well as the requirements for establishing such a relationship of trust and support.

### Conclusion

In this article, we analyzed the relationship between experiences and social representations of sexual transactions based on the data collected from a sample of youth who participated in an online survey. Although we found that most respondents had no experience of sexual transactions, our objective was not to gather details on the conduct of such exchanges. Rather, the main contribution of our analysis has been to shed light on the prevailing norms governing young people’s sexuality in contemporary society. In other words, we set out to explore the understandings, judgments, and positioning of our respondents in relation to sexual transactions. What we found was that young people typically embrace negative social representations of such exchanges as part of an effort to meet social expectations governing sexual behavior.

Our findings run counter to the prevailing wisdom in the academic literature and among professionals, whereby contemporary youth have been adopting increasingly unbridled, risky, or “hypersexualized” behaviors because they lack reference points for sexuality (Blais et al., 2009; Hargot, 2016; Hipeli & Doux, 2009). By questioning this outlook—by highlighting how young people’s expectations regarding sexuality and sexual transactions (whether involving the exchange of money, material goods, or symbolic resources) are very well aligned with social expectations—the results of our analysis contribute to the advancement of both theoretical and professional knowledge. For instance, although sexual socialization has become more about experimentation than the vertical transmission of values, our findings show that sexuality remains tightly framed by social norms that determine the acceptability of specific behaviors. Developed within a heteronormative matrix, these norms are embedded in sexual scripts that exclusively endorse other-sex attraction, and where distinct male and female gender roles are complementary yet asymmetrical.

The results show that social control mainly targets the sexual behavior of young women and young homosexual men. It is important to challenge these preconceptions and social representations of sexual behaviors that fall outside socially established notions of “proper” sexuality. And although a lot of relevant information is available on the Internet, it is important not to overlook the constructive role that adults (especially those within a young person’s circle of trust) can play in helping youth understand their sexuality. In fact, the involvement of adults is key to inspiring social reflection on the dominant models and norms associated with (entry into) sexuality, to learn how to deconstruct them, to promoting recognition of a greater diversity of sexual identities and practices, and to supporting young people in accepting their sexual and gender identity.

## Data Availability

The data that support the findings of this study are available on request from the corresponding author.

## Appendix

See Tables 8, 9, 10.

**Table 8:** Correlations between categories and understandings of sexual transactions

	Social contexts/characteristics		
	Sex work OR (Rob. SE)	Drugs/alcohol OR (Rob. SE)	Internet/smartphones OR (Rob. SE)
**Sexual intercourse (n = 2736)**	0.563***	0.97	1.466*
Gender (woman = 1)	1.400***	1.284***	1.211***
Mid-adolescent	1.293***	1.181*	1.102
Late adolescent	1.397***	1.136	1.279***
Same-sex attraction	0.815	0.700***	0.893
Attraction to both sexes	0.831*	0.709***	0.939
Interactions (second derivatives)	∆2	∆2	∆2
Sexual **intercourse***young women	− 0.168**	− 0.139*	− 0.067
Sexual **intercourse***mid-adolescent	− 0.024	0.046	− 0.072
Sexual **intercourse***late adolescent	0.190*	0.072	− 0.170*
Sexual **intercourse***same-sex	− 0.044	− 0.116	− 0.207*
Sexual **intercourse***both sexes	0.124	0.104	0.036
**Oral sex (n = 2823)**	0.516***	0.876	1.342
Gender (woman = 1)	1.361***	1.257***	1.198***
Mid-adolescent	1.340***	1.196**	1.093
Late adolescent	1.382***	1.123	1.264***
Same-sex attraction	0.890	0.783*	0.911
Attraction to both sexes	0.836*	0.694***	0.980
Interactions (second derivatives)	∆2	∆2	∆2
**Oral sex***young woman	− 0.157**	− 0.075	− 0.011
**Oral sex***mid-adolescent	− 0.050	− 0.032	− 0.074
**Oral sex***late adolescent	0.154*	0.002	− 0.152
**Oral sex***same-sex	− 0.013	− 0.138	− 0.114
**Oral sex***both sexes	0.162**	0.158*	− 0.009
**Showing private body parts (n = 3181)**	0.606***	0.787	1.571**
Gender (woman = 1)	1.365***	1.266***	1.179***
Mid-adolescent	1.276***	1.141*	1.099
Late adolescent	1.387***	1.110	1.287***
Same-sex attraction	0.882	0.771**	0.867
Attraction to both sexes	0.877	0.717***	0.940
Interactions (second derivatives)	∆2	∆2	∆2
**Show private body***woman	− 0.168**	− 0.017	− 0.038
**Show private body***mid-adolescent	0.020	0.100	− 0.024
**Show private body***late adolescent	0.142*	0.111	− 0.099
**Show private body***same-sex	0.016	− 0.102	− 0.035
**Show private body***both sexes	0.092	0.093	0.028

Multivariate ordered probit regression with moderated effects. Odd ratios. 2Δ = second differences for interactions terms (first differences are omitted for brevity as well as standard errors). The estimates for men, early adolescents, and opposite-sex sexual attraction are not shown in the regressions due to perfect collinearity. *** *p* < 0.001; ** *p* < 0.010; * *p* < 0.050

**Table 9:** Correlations between categories and judgments of sexual transactions

	Ethical assessments		
	Appropriate for men or women to offer or accept	Better to offer/accept a gift than money	Less of a problem for the man offers to offer/ the woman to accept
**Sexual intercourse (n = 2736)**	3.246***	2.024***	2.152***
Gender (woman = 1)	0.626***	0.777***	0.750**
Mid-adolescent	0.832	0.832*	0.888
Late adolescent	0.811	0.640***	0.679**
Same-sex attraction	0.863	0.801	1.029
Attraction to both sexes	1.452**	1.033	0.971
Interactions (second derivatives)	∆2	∆2	∆2
Sexual **intercourse***woman	0.166*	0.044	0.156*
Sexual **intercourse***mid-adolescent	− 0.091	− 0.109	− 0.052
Sexual **intercourse***late adolescent	− 0.155	− 0.078	− 0.062
Sexual **intercourse***same-sex	− 0.077	− 0.033	− 0.084
Sexual **intercourse***both sexes	0.113	− 0.144	− 0.091
**Oral sex (n = 2823)**	3.314***	2.030***	2.281***
Gender (woman = 1)	0.614***	0.754***	0.755**
Mid-adolescent	0.867	0.907	0.923
Late adolescent	0.855	0.708***	0.695**
Same-sex sexual attraction	0.865	0.790*	0.986
Attraction to both sexes	1.402**	0.966	1.110
Interactions (second derivatives)	∆2	∆2	∆2
**Oral sex***woman	0.126*	0.036	0.086
**Oral sex***mid-adolescent	− 0.121	− 0.136	− 0.066
**Oral sex***late adolescent	− 0.171*	− 0.161*	− 0.050
**Oral sex***same-sex	− 0.077	− 0.035	− 0.126
**Oral sex***both sexes	0.061	− 0.059	− 0.090
**Show private body parts (n = 3181)**	2.569***	1.702**	2.151***
Gender (woman = 1)	0.611***	0.771***	0.756***
Mid-adolescent	0.883	0.852*	0.908
Late adolescent	0.898	0.681***	0.685***
Same-sex attraction	0.955	0.781*	1.069
Attraction to both sexes	1.329**	0.960	1.088
Interactions (second derivatives)	∆2	∆2	∆2
**Show private body***woman	0.139**	0.065	0.064
**Show private body***mid-adolescent	− 0.108	− 0.019	− 0.092
**Show private body***late adolescent	− 0.197**	− 0.062	− 0.117*
**Show private body***same-sex	− 0.031	0.048	− 0.111
**Show private body***both sexes	0.156*	− 0.060	− 0.057

Multivariate probit regression with moderated effects. Odd ratios. 2Δ = second differences for interactions terms (first differences and SE are omitted for brevity). The estimates for men, early adolescents, and opposite-sex sexual attraction are not shown in the regressions due to perfect collinearity *** *p* < 0.001; ** *p* < 0.010; * *p* < 0.050

**Table 10:** Correlations between categories of sexual transactions and positioning vis-à-vis such exchanges

	Semantic scales		
	Abnormal–normal	Bad–good	Humiliating–respectful
**Sexual intercourse (n = 2736)**	3.098***	3.089***	2.784***
Gender (woman = 1)	0.506***	0.559***	0.542***
Mid-adolescent	0.659***	0.673***	0.736***
Late adolescent	0.587***	0.593***	0.644***
Same-sex attraction	1.238	1.403**	1.215
Attraction to both sexes	1.500***	1.546***	1.183
Interactions (second derivatives)	∆2	∆2	∆2
Sexual **intercourse***woman	9.25*	3.83	5.836
Sexual **intercourse***mid-adolescent	− 12.79*	− 11.57*	− 17.61**
Sexual **intercourse***late adolescent	− 25.48***	− 23.65***	− 21.61**
Sexual **intercourse***same-sex	− 8.89	− 14.10	− 10.86
Sexual **intercourse***both sexes	− 2.85	− 0.049	− 2.506
**Oral sex (n = 2823)**	3.096***	3.130***	2.322***
Gender (woman = 1)	0.530***	0.578***	0.556***
Mid-adolescent	0.708***	0.716***	0.714***
Late adolescent	0.644***	0.656***	0.632***
Same-sex attraction	1.126	1.245*	1.115
Attraction to both sexes	1.221*	1.311**	1.051
Interactions (second derivatives)	∆2	∆2	∆2
**Oral sex***woman	8.20	2.17	3.40
**Oral sex***mid-adolescent	− 10.04*	− 8.96	− 8.14
**Oral sex***late adolescent	− 20.62**	− 24.19***	− 10.73
**Oral sex***Same-sex	− 10.89	− 11.96	5.65
**Oral sex***Both sexes	− 2.37	0.86	2.71
**Show private body parts (n = 3181)**	2.339***	2.449***	2.327***
Gender (woman = 1)	0.517***	0.553***	0.551***
Mid-adolescent	0.688***	0.708***	0.741***
Late adolescent	0.610***	0.636***	0.648***
Same-sex attraction	1.133	1.203	1.031
Attraction to both sexes	1.311**	1.425***	1.113
Interactions (second derivatives)	∆2	∆2	∆2
**Show private parts***woman	9.30	6.18	4.403
**Show private parts***mid-adolescent	− 5.99	− 10.53*	− 12.56*
**Show private parts***late adolescent	− 15.86**	− 22.70***	− 16.84**
**Show private parts***same-sex	− 1.38	− 3.354	5.20
**Show private parts***both sexes	4.58	4.431	− 0.83

Multivariate negative binomial regression with moderated effects. Incidence rate ratios. 2Δ = second differences for interactions terms in nonlinear models (first differences and SE are omitted for brevity). The estimates for men, early adolescents, and opposite-sex sexual attraction are not shown in the regressions due to perfect collinearity. *** *p* < 0.001; ** *p* < 0.010; * *p* < 0.050

## References

[CR1] Averdijk, M., Mueller-Johnson, K., & Eisner, M. (2012). *Sexual victimization of children and adolescents in Switzerland*. UBS Optimus Foundation.

[CR2] Averdijk M, Ribeaud D, Eisner M (2019). Longitudinal risk factors of selling and buying sexual services among youths in Switzerland. Archives of Sexual Behavior.

[CR100] Bajos, N., Ferrand, M., & Andro, A. (2008). La sexualité à l’épreuve de l’égalité. In N. Bajos & M. Bozon (Eds.), *Enquête sur la sexualité en France: pratiques, genre et santé* (pp. 545–576). Paris: La Découverte.

[CR3] Barrense-Dias, Y., Akre, C., Berchtold, A., Leeners, B., Morselli, D., & Surís Granell, J. C. (2018). *Sexual health and behavior of young people in Switzerland* (No. 291). Lausanne, Institut universitaire de médecine sociale et préventive.

[CR4] Benavente B, Diaz-Faes DA, Ballester L (2022). Commercial sexual exploitation of children and adolescents in Europe: A systematic review. Trauma, Violence, & Abuse.

[CR5] Blais M, Raymond S, Manseau H, Otis J (2009). La sexualité des jeunes Québécois et Canadiens Regard critique sur le concept d’« hypersexualisation ». Globe: Revue Internationale D’études Québécoises.

[CR6] Bodmer, N. (2009). Etude sur les attitudes, les connaissances et les comportements des jeunes face à la sexualité. Evolution, influence et perspectives. In CFEJ (Ed.), *La sexualité des jeunes au fil du temps* (7–9). Commission fédérale pour l’enfance et la jeunesse (CFEJ).

[CR7] Boislard M-A, Poulin F, Kiesner J, Dishion TJ (2009). A longitudinal examination of risky sexual behavior among Canadian and Italian adolescents: Considering individual, parental and friend characteristics. International Journal for the Study of Behavioral Development.

[CR8] Bozon M (2012). Autonomie sexuelle des jeunes et panique morale des adultes. Le Garçon sans Frein Et La Fille Responsable. Agora Débats/Jeunesses.

[CR9] Bozon M, Giami A (1999). Les scripts sexuels ou la mise en forme du désir. Présentation de l’article de John Gagnon. Actes de la Recherche en Sciences Sociales.

[CR10] Broqua, C., & Deschamps, C. (2014). *L’échange économico-sexuel*. Éditions de l’École des hautes études en sciences sociales.

[CR11] Bui TC, Nyoni JE, Ross MW, Mbwambo J, Markham CM, McCurdy SA (2014). Sexual motivation, sexual transactions and sexual risk behaviors in men who have sex with men in Dar es Salaam, Tanzania. AIDS and BehavIor.

[CR12] Butler J (1997). Excitable speech.

[CR13] Butler, J. (2006 [1990]). *Gender trouble. Feminism and the subversion of identity*. Routldedge.

[CR14] Carbajal M, Colombo A (2021). Postures professionnelles concernant les transactions sexuelles impliquant des jeunes: Entre souci de (sur)protection et accompagnement de la socialisation sexuelle. Revue Suisse de Travail Social.

[CR102] Carbajal, M., & Colombo, A. (2023). « Encourager les filles à se respecter »: représentations de professionnel∙le∙s accompagnant des jeunes à propos des transactions sexuelles. *Nouvelles Pratiques Sociales, 33*(2), 299–317. 10.7202/1107889ar

[CR15] Carbajal M, Colombo A, Tadorian M (2019). Consentir à des expériences sexuelles sans en avoir envie: La logique de redevabilité: responsabilité individuelle ou injonction sociale genrée?. Journal des Anthropologues.

[CR16] Chauvin S, Lerch A (2013). Sociologie de l’homosexualité.

[CR17] Clair I (2012). Le pédé, la pute et l’ordre hétérosexuel. Agora Débats Jeunesse.

[CR18] Colombo A, Carbajal M (2019). Jeunes et transactions sexuelles médiatisées par le numérique: Échanges indécents ou quête de reconnaissance ?. Revue Suisse De Travail Social.

[CR19] Colombo, A., Carbajal, M., Carvalhosa Barbosa, M., Jacot, C. & Tadorian, M. (2017a). *Sexe, relations… et toi? Sexualité et transactions sexuelles impliquant des jeunes. Synthèse des résultats de recherche*. HES-SO, Haute école de travail social Fribourg (HETS-FR), Fondation Oak. Fribourg : HETS-FR. Retrieved from: www.sexe-et-toi.ch ou www.hets-fr.ch.

[CR20] Colombo A, Carbajal M, Carvalhosa Barbosa M, Tadorian M (2017). Gagner la reconnaissance des pairs en évitant la réputation de «pute». L’injonction paradoxale qui pèse sur les filles impliquées dans des transactions sexuelles. Revue Jeunes et Société.

[CR21] Colombo, A., Carbajal, M., Milani, R., Jacot, C., Baudat, S., Carvalhosa Barbosa, M. & Heeb, J.-L. (2021). *Représentations juvéniles de la sexualité et des transactions sexuelles impliquant des jeunes en Suisse. Rapport des résultats de l’enquête par questionnaire « Sexe, relations… et toi?* » (volet quantitatif). Recherche financée par la Fondation Oak et réalisée par la HES-SO/HETS-FR.

[CR22] Colombo A, Carbajal M, Tadorian M, Lavaud-Legendre B (2022). Les représentations des jeunes à propos de la prostitution: révélatrices d’une morale sexuelle contemporaine. Prostitution de mineures: trouver la juste distance. Prostitutions de mineures. Trouver la juste distance.

[CR23] Combessie P, Mayer S (2013). Une nouvelle économie des relations sexuelles?. Ethnologie Française.

[CR24] Coslin PG (2002). Psychologie de l’adolescent.

[CR25] Crevoisier, O. & Donzallaz, S. (2022). *Sexual economic transactions: Actors, institutions and territories.* Working Paper 3, The Circulation of People, MAPS, University of Neuchâtel.

[CR26] Dayer C (2014). Sous les pavés, le genre: Hacker le sexisme.

[CR27] Debons, J., Lucia, S., Bize, R. (2019). *Etude sur les trajectoires de jeunes LGBTIQ+ confrontés à des expériences d’ordre sexuel associées à un échange financier, matériel et/ou symbolique. Rapport final*. Lausanne, Unisanté—Centre universitaire de médecine générale et santé publique.

[CR28] Delgrande Jordan M, Schneider E, Eichenberger Y, Kretschmann A (2019). La consommation de substances psychoactives des 11 à 15 ans en Suisse. Situation en 2018 et évolutions depuis 1986.

[CR101] Déroff, M. (2007). *Homme/femme: La part de la sexualité. Une sociologie de l’hétérosexualité. Rennes*: Presses universitaires de Rennes.

[CR29] Félonneau ML, Lannegrand-Willems L (2005). Normes adolescentes, normes adultes. Percevoir et juger les incivilités urbaines. Bulletin de Psychologie.

[CR30] Flament C, Jodelet D (1989). Structure et dynamique des représentations sociales. Les représentations sociales.

[CR31] Fredlund C, Svensson F, Svedin CG, Priebe G, Wadsby M (2013). Adolescents’ lifetime experience of selling sex: Development over five years. Journal of Child Sexual Abuse.

[CR32] Gagnon JH, Simon W (1973). Sexual conduct: The social sources of human sexuality.

[CR33] Galland O, Bréchon P (2003). L’évolution des valeurs des Français s’explique-t-elle par le renouvellement des générations?. Les valeurs des Français.

[CR34] Ha T, Kim H, Christopher C, Caruthers A, Dishion TJ (2016). Predicting sexual coercion in early adulthood: The transaction among maltreatment, gang affiliation, and adolescent socialization of coercive relationship norms. Development and Psychopathology.

[CR35] Hargot T (2016). Une jeunesse sexuellement libérée (ou presque).

[CR36] Hipeli S, Doux D, Dans CJ (2009). Génération porno: épouvantail médiatique ou fait?. La sexualité des jeunes à travers les âges: changements, influences, perspectives.

[CR37] Homma Y, Nicholson D, Saewyc EM (2012). A profile of high school students in rural Canada who exchange sex for substances. Canadian Journal of Human Sexuality.

[CR38] Jodelet D (1989). Représentations sociales: Un domaine en expansion. Les Représentations Sociales.

[CR39] Karsz S (2004). Pourquoi le travail social? Définition, figures, clinique.

[CR40] Kohlberg L, Goslin GA (1969). Stage and sequence: The cognitive-developmental approach to socialization. Handbook of socialization theory and research.

[CR41] Lavaud-Legendre B, (dir.),  (2022). Prostitutions de mineures. Trouver la juste distance.

[CR42] Lavaud-Legendre, B. (2022b). L’accompagnement des mineures en situation de prostitution: mission impossible? *Sociétés et jeunesses en difficulté* [Online], *27*. http://journals.openedition.org/sejed/11284.

[CR43] Lavoie F, Thibodeau C, Gagné MH, Hébert M (2010). Buying and selling sex in Québec adolescents: A study of risk and protective factors. Archives of Sexual Behavior.

[CR44] Leclerc-Madlala S (2003). Sexual transactions and the pursuit of modernity. Social Dynamics.

[CR45] Legault G (2000). L’intervention interculturelle.

[CR46] Lemelin C, Lussier Y, Sabourin S, Brassard A, Naud C (2014). Risky sexual behaviors: The role of substance use, psychopathic traits, and attachment insecurity among adolescents and young adults in Quebec. Canadian Journal of Human Sexuality.

[CR47] Lindqvist A, Sendén MG, Renström EA (2021). What is gender, anyway?: A review of the options for operationalising gender. Psychology & Sexuality.

[CR48] Lynch, E., McGovern, R., Elzerbi, C., Breckons, M., Deluca, P., Drummond, C., & Kaner, E. (2019). Adolescent perspectives about their participation in alcohol intervention research in emergency care: A qualitative exploration using ethical principles as an analytical framework. *PLoS ONE,**14*(6). 10.1371/journal.pone.0217855 10.1371/journal.pone.0217855 PMC6561559 31188852

[CR49] Makhakhe NF, Lane T, McIntyre J, Struthers H (2017). Sexual transactions between long distance truck drivers and woman sex workers in South Africa. Global Health Action.

[CR50] Medeiros M, Forest B, Öhberg P (2020). The case for non-binary gender questions in surveys. Political Science & Politics.

[CR51] Megaputri PS (2020). Social media as a tool of sexual transactions of men who have sex with men (MSM) in Buleleng Regency, Bali. Journal Ners Dan Kebidanan Indonesia.

[CR52] Mercier E (2018). Humiliation, responsabilisation et moralisation dans les discours sur le partage l’images intimes chez les jeunes. Revue Jeunes et Société.

[CR53] Mize TD (2019). Best practices for estimating, interpreting, and presenting nonlinear interaction effects. Sociological Science.

[CR54] Narring, F., Tschumper, A., Inderwildi Bonivento, L., Jeannin, A., Addor, V., Bütikofer, A., ... Michaud, P.-A. (2002). *Santé et styles de vie des adolescents âgés de 16 à 20 ans en suisse*. Lausanne, Institut universitaire de médecine sociale et préventive.

[CR55] O’Sullivan LF, Thompson AE, Tolman D, Diamond LM, Bauermeister JA, George WH, Pfaus JG, Ward L (2013). Sexuality in adolescence. Handbook of sexuality and psychology.

[CR56] Parazelli, M., Bellot, C., Gagné, J., Morin, R., & Gagnon, E. (2013). *Les enjeux du partage de l’espace public avec les personnes itinérantes et sa gestion à Montréal et à Québec: perspectives comparatives et pistes d’actions*. Université du Québec.

[CR57] Payne E, Smith MJ, Rodriguez NM, Martino WJ, Ingrey JC, Brockenbrough E (2016). Gender policing. Critical concepts in queer studies and education.

[CR58] Pheterson G (1993). The whore stigma: Woman dishonor and man unworthiness. Social Text.

[CR59] Rémy J, Voyé L, Servais E (1978). Produire ou reproduire? Une sociologie de la vie quotidienne.

[CR60] Ritchwood TD, Ford H, DeCoster J, Sutton M, Lochman JE (2015). Risky sexual behavior and substance use among adolescents: A meta-analysis. Children and Youth Services Review.

[CR61] Sanci LA, Sawyer SM, Haller DM, Patton GC, Kang MSL (2005). Confidential health care for adolescents: Reconciling clinical evidence with family values. Medical Journal of Australia.

[CR62] Schurmans MN (2013). Négociations et transactions: Un fondement socio-anthropologique partagé. Négociations.

[CR63] Svensson F, Fredlund C, Svedin CG, Priebe G, Wadsby M (2013). Adolescents selling sex: Exposure to abuse, mental health, self-harm behavior and the need for help and support. A study of a Swedish national sample. Nordic Journal of Psychiatry.

[CR103] Tabet, P. (2004). *La grande arnaque*. L'Harmattan, Paris.

[CR64] van de Walle R, Picavet C, van Berlo W, Verhoeff A (2012). Young Dutch people’s experiences of trading sex: A qualitative study. Journal of Sex Research.

[CR65] Young AM, McCabe SE, Boyd CJ (2007). Adolescents’ sexual inferences about girls who consume alcohol. Psychology of Women Quarterly.

[CR66] Zelizer V (1989). The social meaning of money: “Special monies”. American Journal of Sociology.

[CR67] Zimmermann G, Barbosa Carvalhosa M, Sznitman G, Van Petegem S, Baudat S, Darwiche J, Antonietti J, Clémence A (2017). Conduites à risque à l’adolescence: Manifestations typiques de construction de l’identité?. Enfance.

